# The influence of negative response style on survey-based household inflation expectations

**DOI:** 10.1007/s11135-015-0161-9

**Published:** 2015-01-25

**Authors:** Piotr Białowolski

**Affiliations:** Institute of Statistics and Demography, Warsaw School of Economics, Ul. Madalińskiego 6/8, 02-513 Warszawa, Poland

**Keywords:** Inflation expectations, Latent class analysis, Survey response styles, C32, E31, E37

## Abstract

The study identified a sub-group of respondents adopting a negative response style in consumer tendency surveys and investigated their influence on aggregate household inflation expectations. Households prone to negative response style were identified using multi-group latent class models. The data source was the State of the Household Survey, conducted following European Commission methodology, in Poland between 1999 and 2010 (45 quarters). Although group size for households with negative response style was shown to fluctuate, negative response was comparable between periods. Micro-level information on response style was used to correct inflation expectations by the creation of additional factors for respondent weights. After compensation: (1) respondent inflation expectations proved more consistent with professional forecasts; (2) there was significantly better correlation between inflation expectations and consumer confidence; (3) compensated inflation expectations demonstrated the Ball–Friedman hypothesis; whereas, this pattern did not emerge for uncorrected data. Of the available household characteristics, income and age were the only significant determinants for negative response style.

## Introduction

The initial purpose of consumer tendency surveys was to collect information on respondents’ current and future (expected) actions (Katona 1946, 1947) and to predict general economic evolution. Reliability of survey based information about economic concepts is, however, very often questionable (see, Bertrand and Mullainathan 2001), which poses an obstacle to its application in forecasting. Most authors argue that household inflation expectations are inferior to professional forecasts (e.g., Ang et al. 2007; Scheufele 2011). Nevertheless, at an aggregate level, they are often found to be a significant predictor for future inflation (see Białowolski et al. 2014). The main evidence for their inferiority is prevalence of various types of bias in survey-based household prediction (Dias et al. 2008; Forsells and Kenny 2002; Mitchell and Weale 2007; Zarnowitz 1992). In this study, we hypothesise that bias results from a household tendency to reflect economically unsound, negative opinions, particularly in times of weak economic performance.

Lamla and Maag (2012) offer one possible explanation for these tendencies; namely, households are rather passive in acquiring information about inflation. Since they do not actively search for information, they are prone to influence from external factors, e.g., mass media. Another explanation can be found in Bruine de Bruin et al. (2012) who show that some consumers are sensitive to the wording of questions in their assessment of inflation expectations and can generate very high estimates in the assessment of “prices in general” rather than “inflation”. This finding is important in the case of the standardized European Commission questionnaire (European Commission 2006) in which inflation expectations are measured according to questions referring to “consumer prices” rather than to more depersonalized “inflation”. This might result in bias in the assessment of economic phenomena, rendering household forecasts less reliable.

Although there is a broad literature on response styles and influence of respondents characteristics on the response styles, to the best of our knowledge they have never been investigated in tendency surveys. Baumgartner and Steenkamp (2006) present four types of bias in social surveys: socially desirable response, acquiescence, mid-point and extreme response styles. Negative response bias, elaborated in this article, might be considered an anti-acquiescence response style with acquiescence defined as a “tendency to agree rather than disagree with items, regardless of item content” (Van Herk et al. 2004).

The need to learn more about genuine (i.e., non-biased) household inflation expectations led to a set of questions addressable only using individual level data:

1. Are there any distinct styles of response to the consumer tendency questionnaire, and if there are, how many?
2. Can one of these styles be called negative?
3. Does a negative response style significantly influence information from responses to the question on inflation expectations?
4. Are the inflation expectations, after compensation for the negative response style, more consistent with those of professional forecasters and more in line with consumer confidence?
5. Is negative response style influenced by certain household characteristics?

These questions have not gained sufficient attention in past studies but are important in the assessment of inflation expectations provided by households.

The differences between expectations obtained from household opinions and surveys of professionals are presented in Sect. 2, which describes the evolution of inflation and inflation expectations in Poland, offering a more detailed explanation for the subsequent analysis. Section 3 deals with the presentation of multi-group latent class models, the method used to identify common survey patterns. Section 4 describes the specification and estimation of the latent class model used to measure response styles in consumer tendency surveys. Specifically, the set of chosen indicators is used initially to check the number of distinct groups during all periods of analysis and then to confirm that negative response style is time-invariant using multi-group latent class analysis. Secondly, covariates are identified to help depict household characteristics associated with the probability of a negative response style. The class membership probabilities obtained serve as an additional factor for respondent weights to determine the probability of a biased response given the response pattern of a respondent. Finally, to illustrate the influence of negative response style on aggregate information about inflation expectations, individually corrected inflation expectations are tested for coherence with inflation expectations of professionals and consumer confidence.

## Inflation and survey based inflation expectations in Poland

**Fig. 1:**
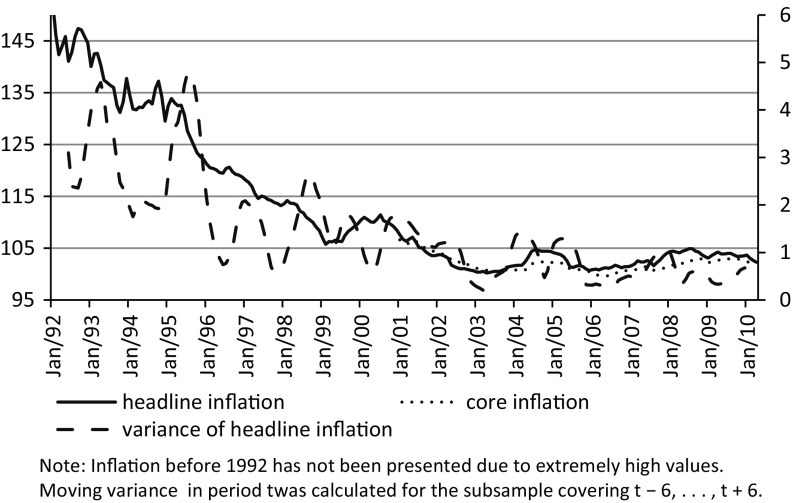
Headline and core inflation (YoY) in Poland between 1992 and 2011. *Source* National Bank of Poland, Central Statistical Office. Note: Inflation before 1992 has not been presented due to extremely high values. Moving variance in period t was calculated for the subsample covering $\hbox {t} - 6, \ldots , \hbox {t} + 6$

For almost a decade after 1989 the Polish economy underwent disinflation. During the period, relatively high levels of inflation along with its high variability, measured by the moving variance, were observed (Fig. 1).

Białowolski et al. (2011), who investigated the process of inflation in Poland, concluded that the relationship between the main macroeconomic variables and inflation was distorted in the period preceding 1999. Additionally, they argue that for the period, it would have been difficult to obtain reliable inflation forecasts and model inflation with inflation expectations. Thus, the period for analysis is limited to cover quarters from the 1st January 1999.

In this paper we argue that respondents might be willing to express their negative attitudes about the economy via the tendency survey questionnaire. The source of individual data for the assessment of this phenomenon was the State of the Household Survey,^1^ conducted at the Research Institute for Economic Development at the Warsaw School of Economics. It is a consumer tendency survey conducted quarterly by mail among a panel sample of Polish households in line with the harmonized questionnaire proposed by the European Commission (2006). The detailed wording of questions is provided by Appendix 1.

In the 1st quarter 1999 (1999Q1)—1st quarter 2010 (2010Q1), 45 periods, an average of 642 households answered the questionnaire each quarter. The minimum number of responses in the given period was 342 and the maximum, 1,552. The questionnaires were sent to households with the assumption that answers were provided by the household head. The results of the survey were post-stratified to match the structure of the population with respect to age and education level of household heads. The problem of missing data was negligible as missing entries for questions subject to the analysis were below 1.1 %.

For Polish households, the result based on aggregates from the State of the Household Survey confirmed overwhelming and unjustified pessimism in the areas of assessment of the household and also the general economic situation. Although during the period 1999–2013 the average rate of GDP growth exceeded 4 %, in the corresponding period on average only 22 % of respondents expected an improvement in the general economic situation on the 12-month horizon, while 51 % predicted possible deterioration. Additionally, considerable pessimism in the area of inflation expectations was observed. Even at times when annual inflation was below 2 % (2002, 2013), about half of those surveyed complained about significant increases in consumer prices. It might be hypothesised that some households adopt a negative response style owing to pessimism with respect to their financial (or economic) position, want to express their anxiety or simply have little knowledge about the forces behind inflation. Although analysis of psychological motives behind negative response patterns is beyond the scope of the article, we argue that the data should be adjusted for this pattern to obtain a more reliable indication of inflation expectations.

**Fig. 2:**
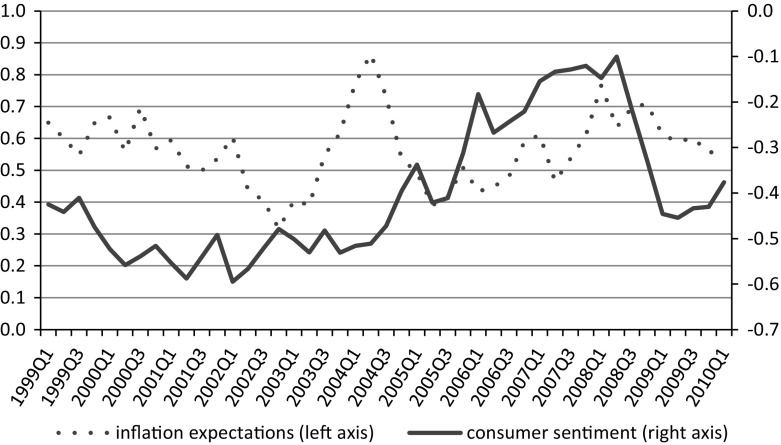
Balances for consumer inflation expectations and confidence calculated in line with the European Commission methodology. *Source* Research Institute for Economic Development – Warsaw School of Economics

Overall pessimism in consumer tendency survey response is likely to yield findings contradictory to economic stylized facts. Economic theory suggests a strong positive relationship between the development of the general economy and changes in the level of inflation, especially in the short term. Economic models show that most short-term fluctuations can be attributed to demand-side shocks (Smets and Wouters 2005, p. 171). Demand-driven changes to the economic environment imply that higher economic growth is associated with higher inflation. Consumer surveys are designed to reflect opinions from the demand side of an economy on matters such as major purchases or the financial situation. Thus, following this line of argument, consumer confidence, as measured with tendency survey questionnaire, understood as the certainty that the economy will develop in a positive direction, i.e., result in greater production, GDP or consumption (Białowolski 2014)^2^ and inflation expectations, should be linked in a positive relationship. However, only a very weak link was detectable from consumer survey data (Fig. 2).

**Fig. 3:**
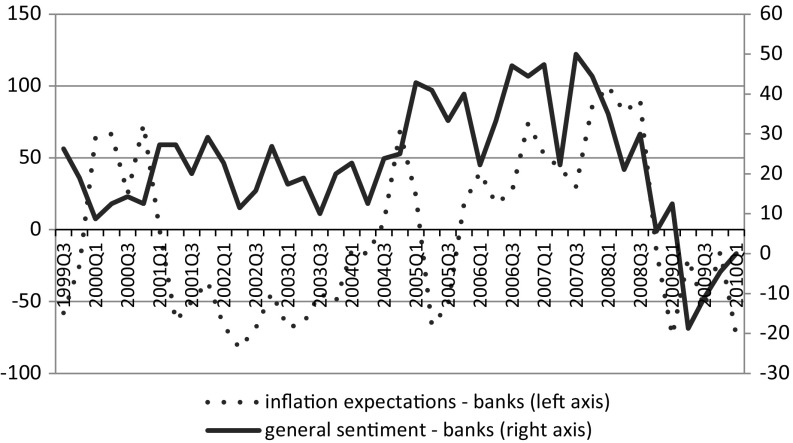
Inflation expectations and forecasts of the general economic situation according to professional respondents in the banking sector (12 months horizon). *Source* Research Institute for Economic Development – Warsaw School of Economics

The correlation coefficient between the two series for the reported time span (1999Q1–2010Q1) is 0.038, not significant with a p value of 0.80. This implies the lack of a linear relationship between consumer confidence and the inflation expectations of households. The opposite conclusion can be drawn from the opinions of banks, an important group of professional forecasters of the Polish economy. According to the results of the “Business Situation in the Banking Sector in Poland”, a business survey conducted in the banking sector, a positive relationship exists between expectations about the general economic situation and expected changes in prices. This relation is given in Fig. 3.

The correlation coefficient is 0.351 with p value 0.02, which shows that professionals consider demand factors important in forming inflation expectations. It might be interpreted from the results based on aggregate data that respondents in the consumer survey do not appreciate that higher inflation and higher growth are correlated (i.e., demand-related processes) and forget or are unaware that a better business climate stimulates inflation.

However, this observation might be only partially true if there is a group of respondents with biased answers distorting the full perspective. In the State of the Household Survey, the presence of a sizeable group of respondents with negative opinions might be found. Among respondents participating in the survey in 2010, those selecting the most extreme negative answers to questions on the prospects of general economy (GES.F) and price forecasts (PRA.F) accounted for more than 15.5 %, while in the survey of banks during the same period, respondents with a similar response style, only 2 %. These differences seem even larger considering that responses to the consumer questions were measured on a five point scale (see Appendix 1 for question wording) and those of banks were measured on a three point scale. Random probability of selecting such a negative response style is only 0.04 in the case of consumer survey and ca. 0.11 in the bank survey. Hence, a much higher likelihood of bias among consumers clearly requires further investigation.

## Method and modelling strategy

For the assessment of response styles in business and consumer tendency surveys a technique is needed to allow decomposition of a response into two parts, one associated with response style and the other to the underlying economic concept. One technique for this decomposition is multi-group latent class analysis. Using this approach, relationships between discrete indicator variables (questions) and the latent variable (class membership) can be modelled. A characteristic feature of latent class analysis is that the latent variable is also discrete and thus defines groups of individuals who express a certain pattern of response. Given that in the analysis, the whole frequency table is analysed in a way not limited to correlations, as in factor analysis, it offers a tool suitable for detection of extreme patterns in survey data (see e.g. Moors 2003).^3^

Multi-group latent classes were originally developed for the analysis of latent structures of categorical latent variables between different numbers of groups (Kankaraš et al. 2011). In our case, different groups correspond to different cycles of the survey. Multi-group latent class models can be described by the set of following assumptions. With $N$ manifest variables $A_{1}\,A_{2} \ldots . A_{N}$ (answers to questions), which each have $M_{i}\, (m_{1} =1..M_{1};\,m_{2}=1..M_{2}; \ldots ; m_{N}=1..M_{N})$ answer categories, one latent variable $X$ with $k=1,\ldots ,K$ classes and one grouping variable $T$ with $t=1,\ldots ,L$ groups, it is possible to define $L$ cross-tables each with $N$-dimensions that represent interrelations between the manifest variables for each group (in our case the time of each survey). Inclusion of the latent variable $X$ leads to the following form of the model:

1 $$\begin{aligned} \pi _{m_1 m_2 \ldots m_N kt}^{A_1 A_2 \ldots A_N X|T} =\pi _{kt}^{X|T} \pi _{m_1 kt}^{A_1 |XT} \pi _{m_2 kt}^{A_2 |XT} \ldots \pi _{m_N kt}^{A_N |XT}, \end{aligned}$$

where $\pi _{m_1 m_2 \ldots m_N kt}^{A_1 A_2 \ldots A_N X|T}$ defines the conditional probability for a respondent with the set of answers $(\hbox {m}_{1}, \hbox {m}_{2},\ldots ,\hbox {m}_{\mathrm{N}})$ in period $t$ belongs to the latent class $k$, while $\pi _{kt}^{X|T}$ defines conditional probability of belonging to class $k$ given period $t$ and $\pi _{m_i kt}^{A_i |XT}$ defines the probability of providing answer $m_{i}$ to item $A_{i}$ given class membership ($k$) and period of analysis (t). Latent class models in such specifications are based on the assumption of local independence, implying that answers to manifest questions ($A_{1}, A_{2}, \ldots , A_{N}$) are mutually independent, given the latent class $k$. Thus, the number of latent classes defines the number of independent answer patterns with that given dataset.

Conditional probabilities $\pi _{m_i kt}^{A_i |XT}$ in a latent class model can be specified and estimated with logistic parameterisation. In such cases, the probability of providing a given answer can be defined as follows:

2 $$\begin{aligned} \pi _{m_i kt}^{A_i |XT} =\frac{e^{thresh_{m_i ,k,t} }}{1+e^{thresh_{m_i ,k,t} }}-\frac{e^{thresh_{m_{i-1} ,k,t} }}{1+e^{thresh_{m_{i-1} ,k,t} }}, \end{aligned}$$

where $M_{i-1} \! \cdot \! K {\cdot } L$ thresholds are estimated for each question and $\forall _{k\in K\wedge t\in L} thresh_{0,k,t}\! =\!-\infty$ and $\forall _{k\in K\wedge t\in L} thresh_{M_i ,k,t} =+\infty$ are given. The probability of latent class membership ($\pi _{kt}^{X|T}$) is estimated in the form of a multinomial logistic regression

3 $$\begin{aligned} \pi _{kt}^{X|T} =\frac{e^{{thresh}_{k,t} }}{1+\sum \limits _{i=1}^{K-1} {e^{thresh_{i,t} }}}. \end{aligned}$$

With multi-group specification it is possible to verify different levels of measurement invariance. Measurement invariance implies that the segmentation rule is the same at all times, which is crucial for comparison between segments at different times. It can be defined on two levels. The most basic multi-group latent class model with measurement invariance assumes equality of thresholds for the probabilities of question answers, which can be formally stated as: $\forall _{i\in N;m_i \in M_i ;k_1 ,k_2 \in K;t_1 ,t_2 \in L} thresh_{m_i ,k_1 ,t_1 } =thresh_{m_i ,k_2 ,t_2}$. This level of measurement invariance is sufficient to ensure structural equivalence of the model (McCutcheon 2002), which takes the form:

4 $$\begin{aligned} \pi _{m_1 m_2 \ldots m_N kt}^{A_1 A_2 \ldots A_N X|T} =\pi _{kt}^{X|T} \pi _{m_1 k}^{A_1 |X} \pi _{m_2 k}^{A_2 |X} \ldots \pi _{m_N k}^{A_N |X} \end{aligned}$$

With this specification, indicator variables—questions—are not directly dependent on the grouping variable (time). The meaning of latent classes, as expressed by indicators (questions), is invariant of the grouping variable. At this level of measurement invariance, a change in the probability of answering to a given question depends only on latent class membership, which can vary at different times. Such a model may be described as partially homogeneous (Kankaraš et al. 2011).

A higher level of measurement invariance is obtained in completely homogenous specifications. This level of measurement invariance requires that the probabilities of class membership are constrained as equal between groups. At this level, formal definition of measurement invariance requires $\forall _{k\in K;t_1 ,t_2 \in L} thresh_{k,t_1 } =thresh_{k,t_2 }$, and the model formally presented as follows:

5 $$\begin{aligned} \pi _{m_1 m_2 \ldots m_N kt}^{A_1 A_2 \ldots A_N X|T} =\pi _{m_1 m_2 \ldots m_N k}^{A_1 A_2 \ldots A_N X} =\pi _k^X \pi _{m_1 k}^{A_1 |X} \pi _{m_2 k}^{A_2 |X} \ldots \pi _{m_N k}^{A_N |X} \end{aligned}$$

This implies that the probability for a given answer set does not depend on the grouping variable (time). The flexibility of multi-group latent class specification also allows inclusion of covariates as predictors of latent class membership. It is given by:

6 $$\begin{aligned} \pi _{kt}^{X|T} =\frac{e^{thresh_{k,t} +\sum \limits _{j=1}^J {\alpha _{j,k} \cdot x_j } }}{1+\sum \limits _{i=1}^{K-1} e^{thresh_{i,t} +\sum \limits _{j=1}^J {\alpha }_{j,k} \cdot x_j }}, \end{aligned}$$

where $\left\{ {x_1 ,\ldots ,x_J } \right\}$ is a set of explanatory variables while $\alpha _{j,k}$ represents estimated parameters that are set equal to zero for one reference class of a covariate.

In the multi-group approach, comparison between models and appropriate selection can either be based on the absolute fit, as defined by tests of likelihood-ratio chi-square ($L^{2}$) and Pearson’s chi-square ($\chi ^{2}$), or on the information criteria (see, e.g., McCutcheon 2002). With respect to the $L^{2}$ and $\chi ^{2}$ tests of absolute model fit, there is controversy concerning ability to deal with sparse tables which are a very common feature of latent class models. These tests reject models too often, while possible flaws might be associated with the missing chi-square distribution for the p value owing to the low number of individuals in a given cell of a sparse table (Kankaraš et al. 2011). Additionally, with a large number of observations, absolute fit tests tend to be too rigorous and reject plausible models.

A commonly adopted approach is to conduct model comparison, selecting the optimal number of latent classes according to information criteria. In this paper, an approach based on the Bayesian Information Criterion (BIC) is proposed (Schwarz 1978) along with the Vuong–Lo–Mendell–Rubin test (Lo et al. 2001; Vuong 1989) to confirm results based on BIC. To check for measurement invariance, the following procedure is adopted: (1) the optimal number of groups is separately established in the model for each period; (2) the number of latent classes in the all-period model is selected, based on the largest number of latent classes that have plausible interpretation in a single period model; and (3) the final solution is tested according to whether the information criteria advocates superiority of the constrained solution versus the unconstrained. With such an approach, it is possible to determine whether heterogeneous, partially homogeneous or completely homogeneous models should be adopted for further analysis.

In the next step, the probability, p, of membership in the latent class characterized by negative response style is calculated for each respondent in each period. This probability is then applied in the calculation of an adjustment factor for the original weight. Original weight is multiplied by (1$-$p). Such an approach builds on the assumption that a given (in our case, negative) response style is viewed as another stage for selection of respondents and the final sample should consist of those not exhibiting negative response style.

For the analysis of factors influencing the latent class membership we first test whether the results obtained with respect to the full sample (1999Q1–2010Q1) for the multi-group latent class model without constraints, also hold for a shorter sample (2005Q1–2010Q1), when all household characteristics of interest in the investigation of negative response style were measured and a panel sample was maintained. In the second step, all household characteristics are introduced to the model as covariates and those with the largest p values are eliminated until only indicators with p values of lower than 0.05 remain.

## Results

### Selection of indicators for the analysis

The consumer tendency questionnaire surveys four fields. It contains items for the assessment of: (1) the present economic situation in the country; (2) the future situation in the country; (3) the current household situation and (4) the future situation of the household. Content and wording of items in each field vary with respect to the underlying economic content, which results in increased chance of detection of any peculiar response mode within the scope of a given field.

To unify the area from which the indicators were taken, first, a set of questions related to perspectives on the economy (GES.F, UNEMP.F, PRA.F) and the current climate (MP.S, SAV.S) was considered (for detailed wording see the Appendix 1). This set of five indicators comprises all questions from the consumer questionnaire that are related to the situation outside the household and oriented towards prediction (European Commission 2006), not past assessment. Second, following the suggestion of Greenleaf (1992), indicators included in the analysis should not be strongly related, i.e., we assume that they are not strongly correlated, in order to avoid any confusion between the particular response style in the set of questions and the underlying economic content of indicators. Therefore, in this analysis it was assumed that the correlation coefficient between the balances^4^ calculated for each indicator, according to EC methodology should not be significant at a level of 0.05. As 45 periods (1999Q1–2010Q1) were included in this analysis, it implied that the threshold value for the correlation coefficient was established at a level of 0.294. The analysis showed that the highest correlation coefficient was between GES.F and UNEMP.F at 0.441, while the second largest correlation coefficient (GES.F and SAV.S) was 0.287. Thus, the question on unemployment was excluded from the analysis. The remaining four questions comprised a mixture of indicators that complied with the assumption of theoretical concepts not closely related, as confirmed additionally by the, at most modest, correlation coefficients.

### Measurement invariance

Following the procedure to detect response patterns presented in point 3, latent class models were estimated separately in 45 cycles of the surveys (1999Q1–2010Q1).^5^ In the process, it was established that in 43 out of 45 cycles, the best-fitting model with respect to the BIC was the model with two latent classes. In the remaining two cases, the best model was one with three latent classes. However, in those two periods the latent probabilities for responses to given questions in the third latent class were not monotonic. Additionally, results of the Vuong-Lo-Mendell-Rubin test supported the two class solution for most periods, offering little support for more than two class solutions (see Appendix 2). As a consequence, a two-class solution was adopted for all periods under analysis and various levels of measurement invariance were checked. Values for BIC for the three specifications of the model with two latent classes are presented in Table 1.

**Table 1:** BIC for heterogeneous, partially homogeneous and completely homogeneous models with two latent classes

	Heterogeneous	Partially homogeneous	Completely homogeneous
BIC	431,670.438	427,564.469	428,532.636

*Source* Own calculations in Mplus

**Fig. 4:**
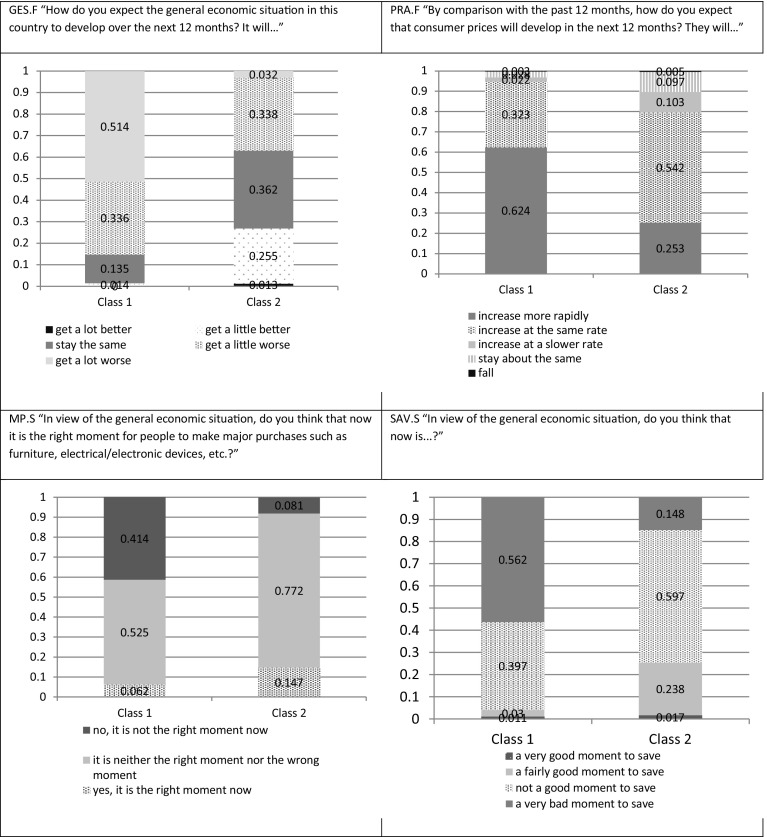
Probability distribution for each of the four indicators conditioned on latent class membership. *Source* Own calculations

The best model appears to be partially homogeneous, with two modes for response comparable between all periods of analysis but with period specific probabilities of being in a given mode. Model-derived conditional response probabilities (given latent class membership) are presented in Fig. 4.

The patterns in class 1 and class 2 are visibly different. Additionally, the pattern in class 1 can be classified as negative. Respondents not only mix responses, indicating high inflation expectations with low levels of expectation about the general economic situation, contradicting demand driven economic processes, but they also report a negative climate with respect to durable goods purchases and saving. Even in the case of expected supply-side-driven inflation there is no justification for such a pattern; for example, in the case of expected high inflation, it would still be profitable to purchase durables now, for which prices are likely to increase. On the other hand, in the case of poor expectations about the general economic situation, there is no economic reason to justify negative evaluation of the climate for savings, because in such a situation contradictory, precautionary motives and negative perspectives for savings are likely to be influential. Grounds for the existence of a group of respondents with negative opinions in all four areas might however be based in a negative response style.

### (In)stability of the proportion of respondents in the negative response style group

**Fig. 5:**
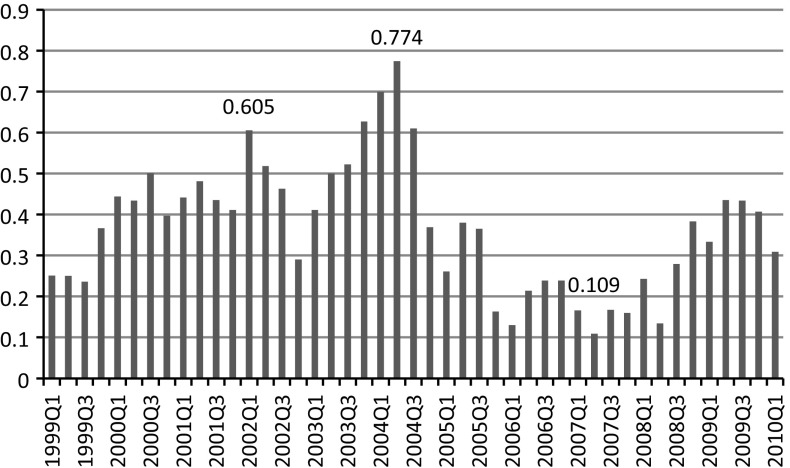
Proportion of respondents characterized by negative response pattern in Poland between 1999Q1 and 2010Q1. *Source* Own calculations

The negative response pattern is not constant in magnitude between periods. Time conditional probabilities of its scale are shown in Fig. 5.

There is a large discrepancy between the proportion of respondents exhibiting a negative response style during different periods, which most likely depends on economic events and media coverage around the time of the survey. The largest share of responses classified in the negative response class was observed before accession of Poland to the EU, when there was widespread conviction that accession would be associated with sudden increase of inflation and additionally, when various parties were trying to convince people that accession might be associated with an economic slowdown. Very high rates for respondents with the negative response style were also observed at the turn of the first decade of the century. In Poland this period was associated with an economic slowdown related to budgetary problems. Nevertheless, the negative response pattern in all areas was not justified since it was a period of very low and stable prices with headline yearly inflation dropping to less than 1 %. Finally, a high percentage of respondents with a negative response pattern was observed after the onset of the financial crisis in the first quarter of 2009.

As the mode of response associated with negative response style contradicts the stylized economic facts, it might be assumed that economic or media events influence public opinion. This would cause significant variation in the proportion of characteristic responses of this type. Therefore they should be excluded from the analysis. It might be that in times of economic turmoil households are more prone to adopt a simple (negative) strategy in responding to the tendency survey questionnaire. It might be also the case that a very large group of respondents is prone to exhibiting dual behaviour, which in times of adverse economic events or media coverage turns into negative response style, while in times of a good economic climate results in moderate response. Such behaviour might be associated with poor understanding of economics. It might also be caused by financial anxiety during economic downturns, which is manifest by negative response to the questionnaire to express negative feelings about the economy. Finally, it might be associated with the overall pessimism of Polish respondents, which is very likely to reveal itself at the downturn of negative economic events.

### Inflation expectations corrected for negative response style

**Fig. 6:**
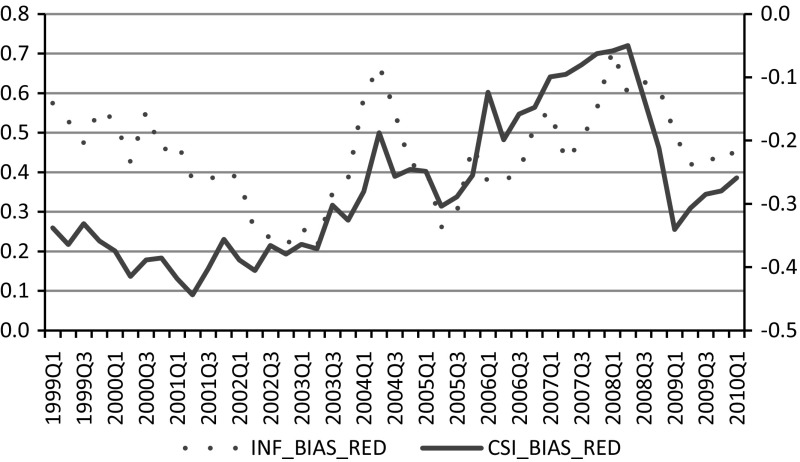
Estimated average values of the consumer confidence index and inflation expectations obtained with the standard balance method after compensation for negative response style. *Source* Own calculations

For each respondent, probability of belonging to a negative response class is implied by the model. The weight obtained during the post-stratification procedure might be multiplied by the probability of not being in the group associated with negative response style, and thus, the weight for the non-negatively biased population can be obtained. These weights are subsequently used to obtain period-specific balances to the questions concerning the inflation expectations, which are presented in Fig. 6.

After compensating for the negative response pattern, the relationship between the average level of consumer confidence (CSI_BIAS_RED) and inflation expectations (INF_BIAS_RED) is significantly altered. Compared to the relation between raw time-series data presented in Fig. 1, the correlation coefficient between the two series increased from 0.038 to a highly significant 0.466 (p value 0.03), in line with expectations for demand-driven inflation. Additionally, household inflation expectations shifted in the direction of inflation forecasts made by professionals (banks). The correlation coefficient between the two time series of balances regarding inflation expectations before the correction for the negative response bias was 0.37 and following correction increased to 0.589.

**Fig. 7:**
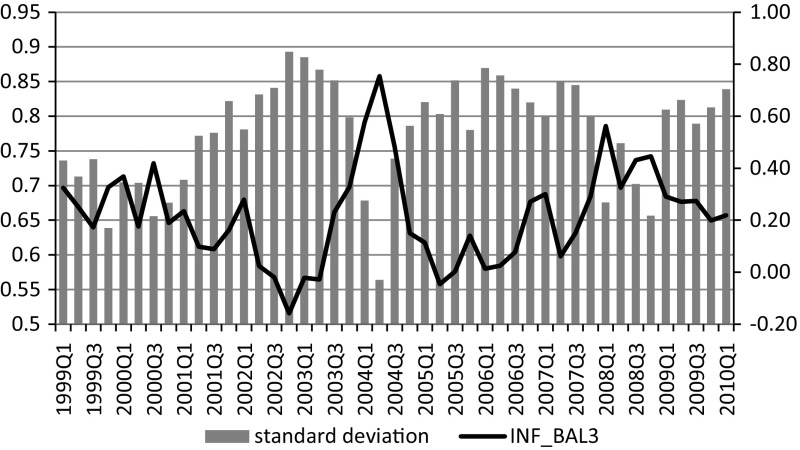
Inflation expectations (average, balance) and variance of inflation expectations. *Source* Own calculations

According to Friedman’s (1977) suggestion, higher average inflation should result in higher inflationary uncertainty, as it distorts relative prices and introduces additional risk to nominal contracts (not adjusted for inflation). This idea was formally proven by Ball (1992) and is currently referred to as the Ball–Friedman hypothesis. Although this idea seems to be intuitive, it is hardly visible in qualitative household predictions for inflation before compensation for negative response style. The relationship between the balance of inflation expectations and its variance (which reflects the disagreement of respondents and can be treated as a proxy for collective uncertainty)^6^ is depicted in Fig. 7.

**Fig. 8:**
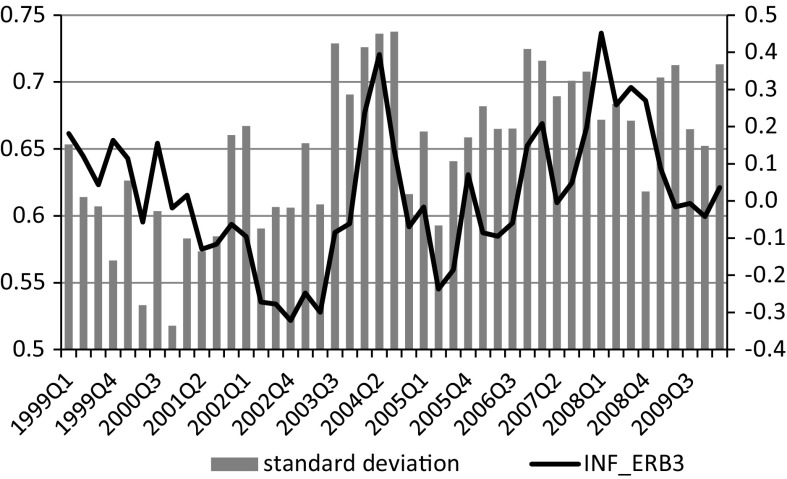
Inflation expectations and their variance after compensation for negative response style. *Source* Own calculations in Mplus based on data from RIED–WSE

The correlation between average inflation expectations and their variance for an index of inflation expectations obtained with the standard balance method is $-$0.78, which indicates that in periods of high inflation expectations, variance of inflation expectations is very low.^7^ However, after correcting for the negative response style, the relation between inflation expectations and their variance is reversed (from $-$0.78 to 0.40). Respondents not expressing a negative response pattern disagree more in their opinions on inflation when they expect it to increase (Fig. 8).

This finding meets the assumptions of the Ball-Friedman hypothesis in which higher inflation expectations are associated with greater inflation uncertainty.

### Negative response style covariates

Baumgartner and Steenkamp (2006) include education level, age and income as among the most important socio-economic covariates of acquiescence and extreme response styles. Following their line of argument, this study investigates whether the expected positive relationship between age and negative response style, as identified in the analysis, and the negative relationship between the other two respondent characteristics and negative response style, also hold for the response mode in the State of the Household Survey. In addition, the probability of negative responses, as associated with the population of place of residence and regularity of participation in the panel, was tested.

Owing to changes in the database for the State of the Household Survey, the examination period was limited to 2005Q1–2010Q1. The results (see Table 2) proved the superiority of the partially homogeneous model over heterogeneous, fully homogenous options and also the shortened sample. Consequently, covariates influencing latent class membership can be meaningfully included in the analysis (see Eq. 6).

**Table 2:** BIC for heterogeneous, partially homogeneous and completely homogeneous models with two latent classes for shortened sample (2005Q1–2010Q1)

	Heterogeneous	Partially homogeneous	Completely homogeneous
BIC	179,735.306	178,404.347	1786,99.807

*Source* Own calculations in Mplus

The analysis included the following covariates:

1. Population of place of residence,
2. Age of respondent,
3. Expected income percentile of household (derived from answers to the question about income on a five-point scale),
4. Total number of completed questionnaires.

Initially, all covariates were included in the analysis and insignificant estimates were eliminated following the backward elimination procedure. The final model is presented in Table 3.

**Table 3:** Logistic regression parameters for membership of the latent class associated with negative response style

	Estimate	Standard error	Odds ratio
Age 16–29	$-$1.125	0.178	0.325
Age 30–49	$-$1.216	0.134	0.296
Age 50–64	$-$0.545	0.107	0.580
Age 65+	Reference		
Income percentile	$-$1.841	0.307	0.159

*Source* Own calculations in Mplus

Respondents aged 16–49 proved much less prone to behave with a negative response pattern. The odds ratio, representing relative probability of a latent class not associated with a negative response style to the probability of a latent class associated with a negative response style, is approximately one third that observed in the group of younger respondents. Even respondents in the age group 50–64 are characterized by over 40 % lower odds to be included in the group with negative response style than those over the age of 64. The negative mode of response to the questionnaire might be perceived as a means of expressing negative opinions about the economy while at the same time conforming with the majority. So, our findings in this domain correspond to Mirowsky and Ross (1991) who claim that acquiescence is positively related to age of respondent.

A second covariate related to membership of the negative response class was household income. The odds ratio for this variable was here, 0.159, indicating that odds for being in the non-negative response class for lowest income households were at less than one sixth of those for highest income households.

## Conclusions

The results presented in this article should prove a suitable starting point for the analysis of response styles in tendency surveys, being coherent with conclusions from studies in various fields. Expression of pessimism about economic perspectives is probably the simplest way of addressing such a tendency survey in Poland. Consumer confidence in Poland has been considerably lower in the past decade than the EU average, while reported GDP growth rates have frequently exceeded those in the EU.

Our results indicate that individual-level data on inflation expectations seem prone to bias. Using a multi-group latent class approach we tested whether a particular group of households answered the set of four selected questions in an illogical, negatively biased way. At almost all times, two latent classes were distinguishable representing negative and moderate response patterns. A partially homogeneous model (with equal meaning of latent classes applied in all periods of analysis) proved the most preferred, both according to the BIC criterion and VLMR test. Thus, the group of respondents demonstrating the negative response pattern can be compared between periods and factors determining class membership for the negative response style group can be meaningfully included in the analysis.

The second major finding is that the indicator of inflation expectations, after accounting for negative response style, provides inflation expectations more consistent with forecasts of professionals. Additionally, expectations prove to be more in line with consumer confidence, which further makes them more reliable on theoretical grounds. Theoretical soundness of the analysis is also confirmed, as the variance of inflation expectations reveals higher uncertainty in times of higher inflation expectations, the hallmark of the Ball-Friedman hypothesis. Otherwise, this was not detectable in raw data. Therefore, the results show that correcting for negative responses in the way suggested might improve the quality of inflation expectations as indicated by household opinion and should perhaps be considered as a first step before forecasts of inflation are made from such survey data.

The results provide very strong arguments for proper selection or at least appropriate weighting of a sample. The analysis of factors influencing negative response style revealed the major importance of age and income as driving forces for negative response style. The group of older respondents and those with lower incomes exhibited a strong prevalence of negative response style. The results also show that even slight overrepresentation of households with lower incomes or those with older members might otherwise lead to a considerable bias associated with negative response style.

## Appendix

See the Tables 4 and 5.

**Table 4:** Standardized consumer tendency survey questionnaire

Question number and code	Question wording	Answer categories (representing also scale points)
Q1 (FS.S)	How has the financial situation of your household changed over the last 12 months? It has...	1.0 “got a lot better”
		2.0 “got a little better”
		3.0 “stayed the same”
		4.0 “got a little worse”
		5.0 “got a lot worse”
		$-$99 “don’t know”
Q2 (FS.F)	How do you expect the financial position of your household to change over the next 12 months? It will...	1.0 “get a lot better”
		2.0 “get a little better”
		3.0 “stay the same”
		4.0 “get a little worse”
		5.0 “get a lot worse”
		$-$99 “don’t know”
Q3 (GES.S)	How do you think the general economic situation in the country has changed over the past 12 months? It has...	1.0 “got a lot better”
		2.0 “got a little better”
		3.0 “stayed the same”
		4.0 “got a little worse”
		5.0 “got a lot worse”
		$-$99 “don’t know”
Q4 (GES.F)	How do you expect the general economic situation in this country to develop over the next 12 months? It will...	1.0 “get a lot better”
		2.0 “get a little better”
		3.0 “stay the same”
		4.0 “get a little worse”
		5.0 “get a lot worse”
		$-$99 “don’t know”
Q5 (PRA.S)	How do you think that consumer prices have developed over the last 12 months? They have...	1.0 “risen a lot”
		2.0 “risen moderately”
		3.0 “risen slightly”
		4.0 “stayed about the same”
		5.0 “fallen”
		$-$99 “don’t know”
Q6 (PRA.F)	By comparison with the past 12 months, how do you expect that consumer prices will develop in the next 12 months? They will...	1.0 “increase more rapidly”
		2.0 “increase at the same rate”
		3.0 “increase at a slower rate”
		4.0 “stay about the same”
		5.0 “fall”
		$-$99 “don’t know”
Q7 (UNEMP.F)	How do you expect the number of people unemployed in this country to change over the next 12 months? The number will...	1.0 “increase sharply”
		2.0 “increase slightly”
		3.0 “remain the same”
		4.0 “fall slightly”
		5.0 “fall sharply”
		$-$99 “don’t know”
Q8 (MP.S)	In view of the general economic situation, do you think that now it is the right moment for people to make major purchases such as furniture, electrical/electronic devices, etc.?	1.0 “yes, it is the right moment now”
		2.0 “it is neither the right moment nor the wrong moment”
		3.0 “no, it is not the right moment now”
		$-$99 “don’t know”
Q9 (MP.F)	Compared to the past 12 months, do you expect to spend more or less money on major purchases (furniture, electrical/electronic devices, etc.) over the next 12 months? I will spend...	1.0 “much more”
		2.0 “a little more”
		3.0 “about the same”
		4.0 “a little less”
		5.0 “much less”
		$-$99 “don’t know”
Q10 (SAV.S)	In view of the general economic situation, do you think that now is...?	1.0 “a very good moment to save”
		2.0 “a fairly good moment to save”
		3.0 “not a good moment to save”
		4.0 “a very bad moment to save”
		$-$99 “don’t know”
Q11 (SAV.F)	Over the next 12 months, how likely is it that you save any money?	1.0 “very likely”
		2.0 “fairly likely”
		3.0 “not likely”
		4.0 “not at all likely”
		$-$99 “don’t know”
Q12 (FIN.S)	Which of these statements best describes the current financial situation of your household?	1.0 “we are saving a lot”
		2.0 “we are saving a little”
		3.0 “we are just managing to make ends meet on our income”
		4.0 “we are having to draw on our savings”
		5.0 “we are running into debt”
		$-$99 “don’t know”

*Source* European Economy (2006), The State of the Households Survey – Research Institute for Economic Development

**Table 5:** Latent class model BIC’s and VLMR test for the periods of analysis

Period of analysis	BIC for unrestricted model (in parentheses p-value for Vuong–Lo–Mendell–Rubin test$^\mathrm{a}$)		
	2 class	3 class	4 class
1999Q1	**11274.203 (0.000)**	11289.962 (0.999)	11359.324 (0.795)
1999Q2	**7618.124 (0.000)**	7669.437 (0.332)	7728.351 (0.671)
1999Q3	**5485.83 **(0.000)	5541.281 **(0.040)**	5605.464 (1.000)
1999Q4	**6927.317 **(0.003)	6949.013 **(0.001)**	7005.36 (0.212)
2000Q1	**5087.85 (0.000)**	5120.928 (0.424)	5188.348 (0.070)
2000Q2	**3868.167 **(0.000)	3926.556 **(0.007)**	3996.393 (0.729)
2000Q3	**2926.118 (0.000)**	2976.711 (0.320)	3044.178 (0.403)
2000Q4	**3068.284 (0.000)**	3133.993 (0.361)	3204.782 (1.000)
2001Q1	**8461.7 **(0.000)	8484.137 **(0.034)**	8541.571 (1.000)
2001Q2	**4006.301 **(0.301)	4044.387 (0.140)	4103.927 (0.483)
2001Q3	**4442.94 **(0.000)	4474.501 **(0.000)**	4537.737 (0.973)
2001Q4	**4636.708 (0.000)**	4687.269 (0.542)	4751.883 (0.221)
2002Q1	**4578.226 (0.000)**	4627.156 (1.000)	4686.891 (0.864)
2002Q2	**3921.068 (0.000)**	3958.77 (0.306)	4013.846 (0.583)
2002Q3	**3531.029 (0.000)**	3580.736 (0.188)	3630.503 (0.526)
2002Q4	**3421.868 (0.028)**	3480.352 (0.936)	3542.132 (1.000)
2003Q1	**4986.481 (0.000)**	5041.731 (0.972)	5105.155 (0.944)
2003Q2	**3652.786 (0.000)**	3714.89 (0.605)	3781.75 (0.247)
2003Q3	**4101.763 (0.000)**	4151.679 (0.599)	4212.834 (0.114)
2003Q4	**3729.019 **(0.062)	3777.646 (0.641)	3836.874 (1.000)
2004Q1	**3752.451 (0.002)**	3815.633 (0.698)	3882.929 (0.625)
2004Q2	**2985.563 (0.000)**	3046.983 (0.417)	3112.514 (0.952)
2004Q3	**3046.338 (0.000)**	3101.247 (0.194)	3158.492 (0.704)
2004Q4	**3241.967 **(0.000)	3287.373 **(0.025)**	3341.3 (0.069)
2005Q1	3873.865 (0.445)	**3856.747** (1.000)	3906.667 (0.765)
2005Q2	**3162.472 **(0.523)	3177.086 (0.530)	3193.04 (0.779)
2005Q3	**2977.665 **(0.171)	3021.231 (0.460)	3068.842 (0.635)
2005Q4	2992.72 (0.871)	**2937.851** (0.302)	2948.449 (0.594)
2006Q1	**3074.441 (0.000)**	3127.747 (0.590)	3199.389 (0.584)
2006Q2	**3525.29 (0.008)**	3581.889 (0.911)	3645.106 (0.218)
2006Q3	**2883.449 **(0.267)	2918.32 (0.639)	2964.948 (0.772)
2006Q4	**2962.5 **(0.734)	3014.27 (0.343)	3075.975 (1.000)
2007Q1	**6868.823 (0.020)**	6919.052 (0.127)	6987.432 (1.000)
2007Q2	**5741.75 **(0.140)	5776.781 (0.501)	5841.045 (0.331)
2007Q3	**5201.341 (0.043)**	5249.586 (0.555)	5311.222 (0.662)
2007Q4	**4898.205 **(0.121)	4910.099 (0.216)	4958.476 (0.761)
2008Q1	**5473.489 **(0.262)	5528.765 (0.761)	5604.326 (0.483)
2008Q2	**5296.928 **(0.583)	5345.685 (0.728)	5399.88 (0.768)
2008Q3	**4697.289 **(0.065)	4731.965 (0.549)	4777.933 (0.467)
2008Q4	**4665.248 **(0.155)	4702.533 (0.763)	4760.38 (0.356)
2009Q1	**4911.825 **(0.098)	4952.82 (0.796)	4996.14 (0.797)
2009Q2	**6300.925 (0.012)**	6338.337 (0.708)	6394.425 (0.282)
2009Q3	**6757.694 (0.037)**	6777.484 (0.603)	6811.032 (0.284)
2009Q4	**5933.787 (0.021)**	5941.557 (0.454)	6000.244 (0.618)
2010Q1	**6598.756 **(0.621)	6628.818 (0.325)	6674.189 (0.000)

*Source* Own calculations in MPlus based on data from RIED WSE

Values in bold represent solutions favoured by the BIC and values in bold and parentheses preferred solution with respect to the VLMR test

$^\mathrm{a}$ Results for Vuong–Lo–Mendell–Rubin Test for k-class solution represent p-value for rejecting H0 that the correct number of classes is k$-$1

## Footnotes

1
http://kolegia.sgh.waw.pl/en/KAE/structure/IRG/Research/Pages/households.aspx.

2 The consumer confidence calculated in line with the European Commission methodology (2006) is performed with the following formula: ${ CCI}\,=\,\frac{{ BAL}_{FS.F} +{ BAL}_{{ GES}.F} +{ BAL}_{{ SAV}.F} -{ BAL}_{{ UNEMP}.F} }{4}$. With respect to the inflation forecasts $BAL_{PRA.F}$ was used. For all the balances calculations were done according to the formula: $BAL\,=\,f_1 +0.5f_2 -0.5f_4 -f_5$, where $\forall _{i\in \{1,2,3,4,5\}} f_i$ stands for the fraction of respondents that selected $i$–th response category for a given question. Refer to the Appendix for detailed wording of questions and response categories.

3 For the detection of bias in social surveys, Morren et al. (2011) suggest an extended approach of Latent Class Factor Analysis. In the case of tendency surveys, where the relation between the indicators is determined by economic theory (negative response style stands against the economic theory) there is no need to introduce the additional factor model.

4 For the formula for calculation of balances see the footnote 2.

5 In the Mplus 6 program in which multi-group latent class is estimated, the number of groups is limited to 45; that is why the sample was shortened to 2010Q1.

6 The current trend is to distinguish between uncertainty at the level of individuals and disagreement associated with the variance of answers among all respondents. It might be associated with the fact that uncertainty concerning the proper model of inflation expectations results in response disagreement. The uncertainty of model selection is discussed in, for example, Branch (2007).

7 Owing to the inverse scale for average inflation expectations obtained in the MGCFA, positive correlation implies higher inflation uncertainty during the periods of low inflation.
